# The prevalence of hepatitis B virus infection in Bangladesh: a systematic review and meta-analysis

**DOI:** 10.1017/S0950268822000061

**Published:** 2022-02-14

**Authors:** Sujan Banik, Anamika Datta, Antara Ghosh, Kanak Yadab Ghosh, Hoimonti Debi

**Affiliations:** 1 Department of Pharmacy, Noakhali Science and Technology University, Noakhali 3814, Bangladesh; 2 Department of Medicine, Dhaka Medical College, Dhaka 1200, Bangladesh

**Keywords:** Bangladesh, hepatitis B, meta-analysis, prevalence

## Abstract

Despite the availability of an effective vaccine, hepatitis B virus (HBV) infection is one of the major public health problems worldwide, mostly in developing countries. This systematic review and meta-analysis were performed to estimate the pooled prevalence of HBV infection in Bangladesh. We systematically searched various electronic databases to retrieve relevant studies published until April 2021. A total of 15 studies were met the inclusion criteria and included in the meta-analysis. The pooled estimated prevalence of HBV infection in the general population of Bangladesh from 1995 to 2017 was 4.0% [95% confidence interval (CI) 3.0–5.1]. The results of subgroup analysis revealed that the prevalence of hepatitis B was higher in females than males [odds ratio (OR) 1.20, 95% CI 0.48–2.97, *P* = 0.70], people of age <25 years had a higher prevalence than people of age >25 years (OR 1.25, 95% CI 0.72–2.17, *P* = 0.42) and married people had a higher prevalence than unmarried/single people (OR 2.16, 95% CI 1.51–3.10, *P <* 0.0001). The Egger's test statistics (*P* = 0.584), Begg and Mazumdar's rank correlation test (*P* = 0.054) indicated the absence of publication bias. This study analysis reported a low intermediate prevalence of HBV infection (4%) in Bangladesh, which is currently higher than the global prevalence of HBV infection (3.5%).

## Introduction

Hepatitis B is a highly infectious disease caused by the hepatitis B virus (HBV). Despite the availability of an effective vaccine, HBV is considered a major public health problem in developing countries [1]. Globally, around 2.57 billion people are estimated to be infected with HBV and among them, more than 250 million are chronically infected with an increased risk of developing HBV-related liver diseases, including liver cirrhosis (LC) and hepatocellular carcinoma (HCC) [2, 3]. According to the global burden of disease, almost 887 000 deaths occurred in 2015 due to acute and chronic HBV infections and 17%, 40% and 43% of deaths were caused by acute infections, LC and HCC, respectively [2]. The prevalence of HBV infection is highly heterogeneous throughout the world and classified as high prevalence areas wherein more than 8% of the people is positive for hepatitis B surface antigen (HBsAg); high intermediate prevalence areas wherein 5–7.99% of the people HBsAg positive; low intermediate prevalence areas wherein 2–4.99% of the people is HBsAg positive; and low prevalence areas wherein less than 2% of the people is positive for HBsAg [4, 5].

Bangladesh is a densely populated developing nation of South Asia, according to the first report in 1984, the prevalence of HBV infection was 7.60% in Bangladesh and indicated as a country with a high intermediate prevalence of hepatitis B [6]. The major difficulties for hepatitis B elimination in Bangladesh, including a lack of reliable data to guide policy, low awareness and barriers to early diagnosis, transmission via infected blood products, unsafe injections and nosocomial transmission and a lack of treatment availability. However, recent studies from 2005 onwards, reported that the prevalence of HBV infection in Bangladesh is at a decline. In 2003, Bangladesh Government included the hepatitis B vaccine in the Expanded Programme of Immunisation (EPI), intending to provide a timely birth dose to newborn babies. It was identified as the most effective HBV immunisation programme across the country with a reported successful HBV vaccination rate of 95% in children [7]. However, the scenario was different for adults, who were not included in the free vaccination campaign that ran from 2003 to the present day in Bangladesh. According to studies conducted in 1991, 1997, 2008 and 2011, the reported prevalence of hepatitis B were 8.6%, 6.4%, 5.5% and 6.5%, respectively in adults [8]. Besides, details are scarce on the prevalence of HBV infection throughout the general community of Bangladesh and no studies have been carried out at the country level.

Therefore, as there is currently no comprehensive study in this regard in Bangladesh, the present meta-analysis was performed to estimate the current prevalence of HBV infection in the general population of Bangladesh based on the data previously published from 1995 to 2021.

## Methods

### Literature search strategy

This systematic review and meta-analysis on the prevalence of hepatitis B in Bangladeshi people were conducted according to the guidelines of Preferred Reporting Items for Systematic Review and Meta-Analyses (PRISMA) [9] (Supplementary file 1). The four international databases (PubMed, Google Scholar, Scopus and Web of Science) and the national databases (Bangladesh Journals Online) were searched for all published studies up to April 2021. The following keywords such as ‘hepatitis B’, ‘hepatitis B virus, ‘HBV’, ‘HBsAg’, ‘prevalence’, ‘prevalent’, ‘epidemiology’, ‘Bangladesh’ and ‘Bangladeshi people’ were used during the search. Additionally, the native journals that could not be accessed online were searched manually and unpublished studies were retrieved from the library of Noakhali Science and Technology University, Bangladesh.

### Inclusion and exclusion criteria

We adopted cross-sectional studies reporting on the prevalence of hepatitis B in the Bangladeshi people. The listed inclusion criteria were the following: (1) study published in a peer-reviewed journal in English; (2) reported studies were accessible in full text (editorial, conference abstracts, case reports not included); and (3) all articles were published from 1995 to 2021. The exclusion criteria were the following: (1) study published in other languages than English; (2) study populations coinfected with HBV, HCV and HIV and (3) study design with non-random sampling like conducted on blood donors, sex workers and connected to other clinical studies.

### Data extraction

The data extraction was conducted independently by three authors (AG, KYG and HD) based on study design and inclusion criteria. After reviewing a full-text article, the following data were extracted from each study including first author name, publication year, year of data collection, study design, sample size, study setting (urban or rural), gender, age, sample size, number of HBsAg positive individuals and HBsAg test methods. Any inconsistencies during the data extraction were solved through careful discussion with other authors (AD and SB).

### Quality assessment

For evaluating the quality of all included studies, further, we assessed the articles using the Cross-Sectional/Prevalence Study Quality Assessment Forms which were suggested by the Agency for Healthcare Research and Quality (AHRQ) [10]. This AHRQ form has eleven questions (Supplementary file 2: Table S1), of which ten questions are fitted for cross-sectional studies, and the questions were answered with ‘Yes’, ‘No’ and ‘Unclear’. ‘Yes’ represented a score of 1 and ‘No’ or ‘Unclear’ represented a score of 0. The total score of ten questions in the AHRQ form was used to assess the quality of each article in this systematic review and meta-analysis.

### Statistical analysis

The Freeman–Tukey double arcsine transformation of proportions was used to estimate the pooled prevalence of HBV infection [11, 12]. DerSimonian-Laird random-effects models were followed to conduct the meta-analysis with a 95% confidence interval (95% CI). We conducted subgroup analyses on the included studies based on age groups, genders and marital status. The existence of heterogeneity across studies was evaluated using both the Cochrane chi-square *Q*-test and *I^2^* index with its resultant *P*-value. A value of *I^2^* 25%, 50% and 75% was used to define the heterogeneity of low, medium and high, respectively. Standard error was estimated from the sample size according to Wan *et al*. [13] method. Finally, the funnel plot was used to assess the possible publication bias graphically and formally by Begg's and Egger's test (significant at *P* < 0.05). All analyses were carried out by MetaXL software (meta-analysis in Microsoft Excel) and Review Manager 5.4 (RevMan 5.4, the Cochrane Collaboration, Oxford, United Kingdom).

## Results

### Search result

**Fig. 1.**
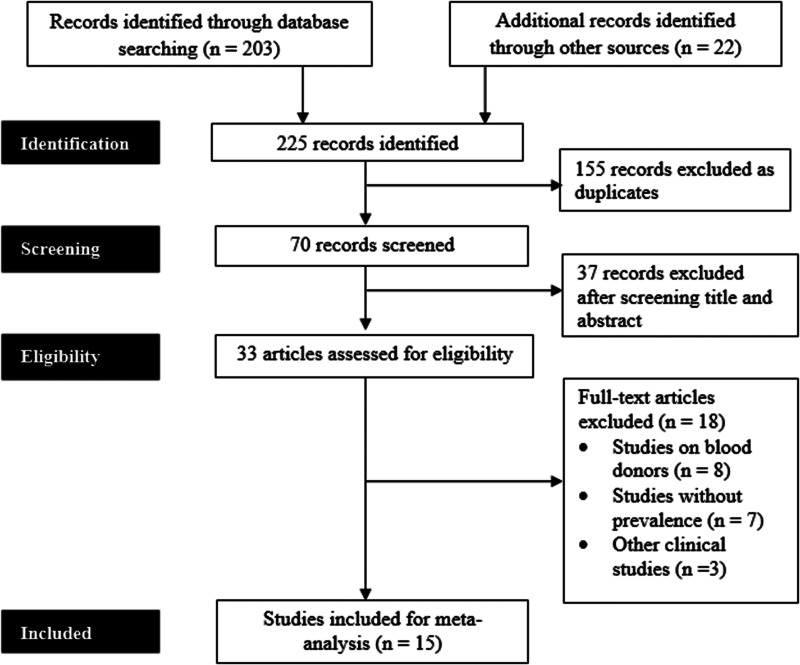
Flowchart showing the literature searching process of the study according to PRISMA guidelines.

A total of 225 articles were identified through various databases searches. After the removal of duplicate records, additional screening and analysis of the titles and abstracts, 33 articles were retrieved for final assessment. Among 33 studies, 15 articles met the eligibility criteria and were finally included in the meta-analysis. Figure 1 shows the process of the literature review, screening and eligibility assessment of the study articles.

### Characteristics of included studies

The included 15 studies with a total sample size of 60 496 reported the prevalence of hepatitis B in Bangladesh and the characteristics of all studies are presented in Table 1. All the included articles [14–28] were cross-sectional studies and conducted throughout the period ranging from 1995 to 2017. The smallest sample size was 130 [14] and the largest sample size was 43 213 [26]. Eight studies were conducted in urban settings [16–19, 21, 25, 26, 28], three in semiurban settings [22, 23, 27], two in rural settings [14, 24] and two studies covered both rural and urban areas [15, 20]. In terms of quality, all studies rated as high quality and had a total quality score higher than 4 (Supplementary file 2: Table S2).

**Table 1.** Characteristics of the studies included in the meta-analysis, order by year of publication and alphabetically within the same year

Author, year of publication	Year of data collection	Study design	Study area	Age group (years)	Sample size (*N*)	HBsAg positive (*n*)	Prevalence of HBsAg (%)	Prevalence of anti-HBc (%)	Diagnostic method	Quality grade
Laskar *et al*. (1997)	1995	Cross-sectional	Urban	11–16	846	7	0.83	–	ELISA	4
Rahman *et al*. (1997)	1994–1995	Cross-sectional	Rural	≥ 15	1000	64	6.4	–	ELISA	4
Rumi *et al*. (2000)	1994–1996	Cross-sectional	Urban	≥ 20	43 213	1884	4.4	–	ELISA	5
Gibney *et al*. (2001)	1999	Cross-sectional	Urban	15–60	388	23	5.9	48.1	ELISA	4
Zaki *et al*. (2003)	1997–1998	Cross-sectional	Urban	0–60	534	16	3.0	21.1	ELISA	5
Alam *et al*. (2006)	2002–2003	Cross-sectional	Rural and urban	0–15	323	23	7.1	–	ELISA	6
Mahtab *et al*. (2008)	2007	Cross-sectional	Semi-urban	16–50	1018	56	5.5	–	ELISA	6
Ashraf *et al*. (2010)	2005–2006	Cross-sectional	Urban	10–38	1997	130	6.5	–	ELISA	5
Mamun-Al-Mahtab *et al*. (2011)	2010	Cross-sectional	Semi-urban	0–60	1018	9	0.88	–	ELISA	6
Rudra *et al*. (2011)	2008–2009	Cross-sectional	Urban	≥ 16	2015	126	6.3	–	ELISA	5
Yasmin *et al*. (2011)	2007	Cross-sectional	Semi-urban	18–25	146	11	7.5	–	ELISA	4
Khan *et al*. (2012)	1995–2012	Cross-sectional	Rural and urban	17–18	1643	5	0.31	–	ELISA	4
Afroz *et al*. (2013)	2012	Cross-sectional	Urban	20–60	3971	194	4.9	–	ELISA	5
Jobayer *et al*. (2016)	2013	Cross-sectional	Urban	18–45	2254	53	2.4	–	ELISA	5
Akhter *et al*. (2017)	2017	Cross-sectional	Rural	NA	130	5	3.9	–	ELISA	4

### Meta-analysis

**Fig. 2.**
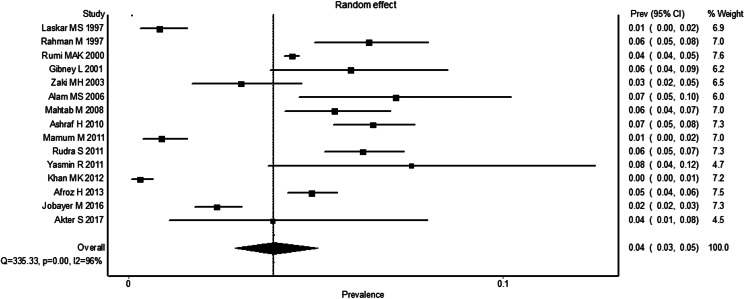
The pooled prevalence of HBV infection in the general people of Bangladesh.

**Fig. 3.**
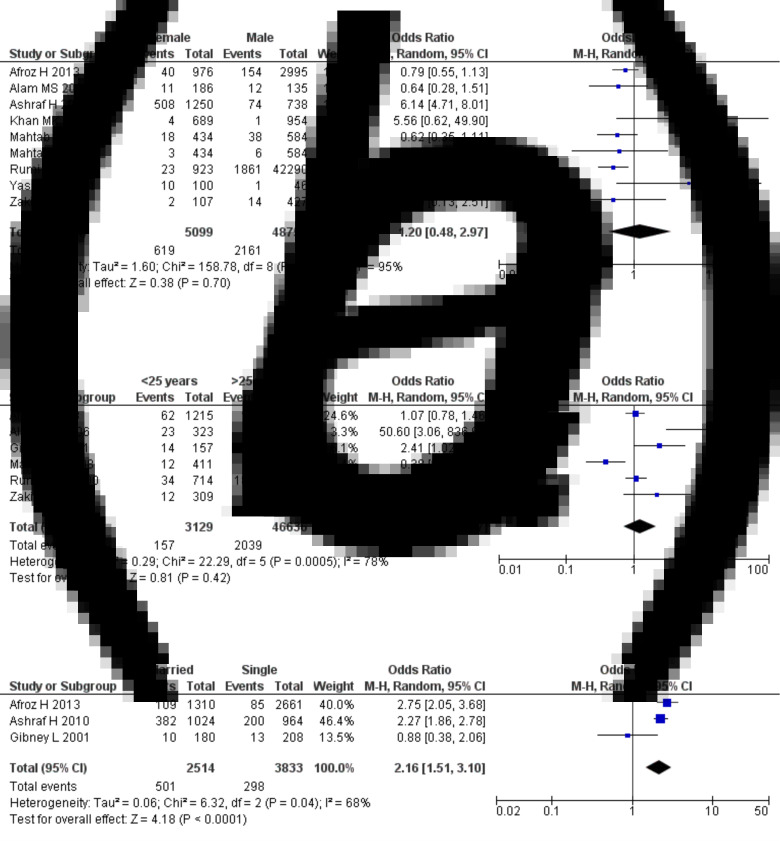
Sub-group meta-analysis reporting HBV prevalence in Bangladesh. (A) between gender, (B) between age groups and (C) between married and unmarried people.

**Fig. 4.**
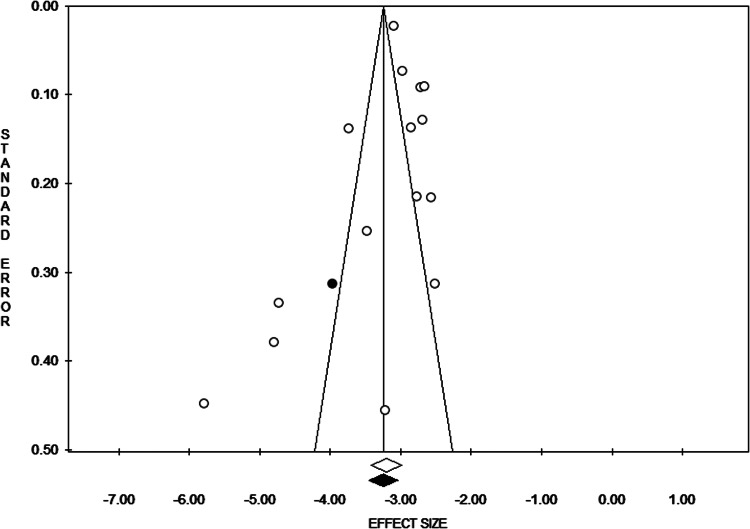
Funnel plot of the meta-analysis.

The pooled estimated prevalence of HBV infection in the general people of Bangladesh was 4.0% (95% CI 3.0–5.1) (Fig. 2). The results of the heterogeneity test showed that the included studies were significantly heterogeneous (*I^2^* = 96%, *P* < 0.0001). Figure 3 shows the results of subgroup analysis of HBV infection among the included studies based on age groups, gender and marital status. The estimated prevalence of hepatitis B was higher in people of age <25 years compared to the people of age >25 years [odds ratio (OR) 1.25, 95% CI 0.72, 2.17, *P* = 0.42] with heterogeneity *I*^2^ 78%. The prevalence of hepatitis B was higher in females in comparison to the males (OR 1.20, 95% CI 0.48, 2.97, *P* = 0.70) with heterogeneity *I*^2^ 95%. The results also demonstrated that the prevalence of hepatitis B was significantly higher in married people than unmarried/single people (OR 2.16, 95% CI 1.51, 3.10, *P* *<* 0.0001) with heterogeneity *I*^2^ 68%. Finally, the funnel plot (Fig. 4) and Egger's linear regression (*P* = 0.584) and Begg and Mazumdar's rank correlation test (*P* = 0.054) for the prevalence of HBV infection indicated that there was no evidence of possible publication bias in all studies.

### HBV infection in Bangladesh over time

**Fig. 5.**
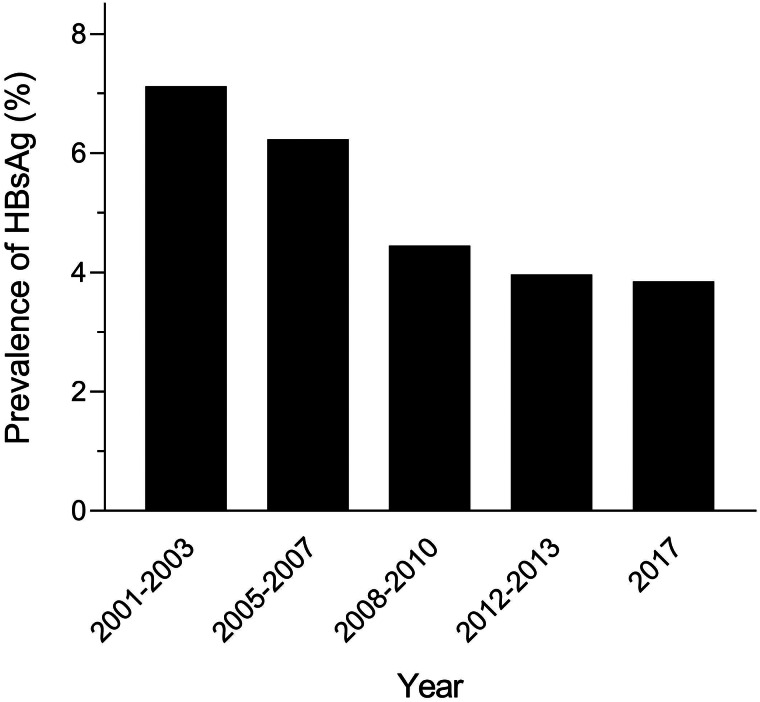
Prevalence trend of HBV infection in Bangladesh over time after the implementation of EPI.

Based on the publications included in the present study, we examine the changes in the reported prevalence of HBV infection in the general population of Bangladesh over time (Fig. 5). According to studies, the prevalence of HBV infection in Bangladesh was 7.12% between 2001 and 2003. In Bangladesh, a decreasing trend in the prevalence of HBV infection was seen after the implementation of EPI, which started in 2003. According to the most current research, which was conducted in 2017, the reported prevalence of HBV infection in Bangladesh was 3.85%.

## Discussion

Worldwide, hepatitis B is a major public health concern and around 3.5% of people are identified as chronic HBV carriers according to a report of 2015 [29]. To obtain the latest data on the prevalence of hepatitis B in the general population of Bangladesh, this systematic review and meta-analysis were performed based upon the published data in the years 1995–2021. According to our systematic review, the pooled prevalence of HBV infection in the general population of Bangladesh was 4% (95% CI 3.0–5.1), which is considered as a low intermediate prevalence rate and indicated a higher prevalence rate in Bangladesh than the global prevalence rate (3.5%). It is also mentioned that a decreasing trend in the prevalence of HBV infection was observed in Bangladesh after the implementation of EPI, which started in 2003 (Fig. 5). Most of the studies (8 studies) included in the present study showed a prevalence rate in the range of 5–8% (high intermediate prevalence), 4 studies which have a range of 2–4% (low intermediate prevalence) and 3 studies have the prevalence rate <2% (low prevalence) (Fig. 2). The scenario of other Asian countries is also variable in most studies. The reported prevalence rates in South Asian countries like Pakistan, Bhutan and Nepal were 2.76%, 5.84% and 0.9%, respectively [2, 30]. Whereas, the prevalence of hepatitis B was higher in South East Asian countries of Thailand (5.1%) and Malaysia (4%) [31, 32]. In the case of comparison with East Asian countries, China has a higher intermediate prevalence rate (6.89%) [33] and Japan has a lower prevalence rate (1.02%) [2]. Therefore, it is confirmed that the prevalence rate of HBV infection varies significantly with countries and regions. The potential causes for this discrepancy could be due to the socio-cultural status, level of awareness and infection prevention practice by the community of different countries. The identified major factors for a moderate prevalence rate of HBV infection in Bangladesh are lack of education, lack of awareness regarding HBV vaccination, limited reliable data to guide policy, poverty, lack of blood screening/cross-checking, perilous sexual activity, vertical transmission from mother to child, costly treatment of HBV infection and lack of proper health care facilities [7, 34–37].

The findings of the research included in this study are very heterogenous, owing to the fact that they were conducted on a variety of different populations and that the screening methods used were inconsistent. However, subgroup analysis was performed to reduce this heterogeneity and provided more details on HBV infection. According to subgroup analysis, we found people of age <25 years had a 1.25 times higher prevalence of HBV infection than the people of age >25 years (OR 1.25, 95% CI 0.72, 2.17, *P* = 0.42). Even though younger individuals had greater vaccine coverage, the majority of children and young people had increased prevalence as a consequence of hepatitis B transmission from mother to baby, sexual activity and, most importantly, illegal drug use via intravenous injection. Recent studies reported that drug addiction is much higher among Bangladesh's young than the elderly population [7, 35]. We found hepatitis B was significantly more prevalent in married people than the unmarried/single people (OR 2.16, 95% CI 1.51, 3.10, *P* *<* 0.0001). Consistent with this study, a higher prevalence of HBV infection was reported in married people in Iran [38] and Pakistan [39] than the unmarried people due to the lack of awareness and education about sexual practices between husband and wife, as HBV is transported through semen and vaginal secretions [40]. The results of subgroup analysis also revealed that the prevalence of hepatitis B was 1.20 times higher in females than males (OR 1.20, 95% CI 0.48, 2.97, *P* = 0.70). Women could be infected due to unprotected sex with her husband or partners and during pregnancy, their modified immune system cannot fight against viral hepatitis load [41]. In contrast, a recent study of China reported the prevalence of HBV infection was higher in males than females (5.88% *vs.* 5.05%) might be due to occupational risk factors, drug usage and male homosexuality [33].

This meta-analysis has the following limitations behind the importance of the study. The prime limitation of this meta-analysis is that most of the articles had a lower number of participants. Secondly, the inconsistency of screening methods might have contributed to the heterogeneity of pooled prevalence estimates. Finally, the information regarding subgroup analysis was only limited to gender, age and marital status, but not included other socio-economic conditions of the participants.

## Conclusion

According to our report, Bangladesh has a low intermediate prevalence rate of HBV infection (4.0%) with an inflated prevalence rate in female, married and younger adults. As the analysis covered vast numbers of studies, it represents the existing prevalence and epidemiology of hepatitis B in the country that could be beneficial for public health leaders to decision-makers. More comprehensive research with larger sample sizes should be conducted to improve the understanding and identification of HBV epidemiology in Bangladesh. In order to achieve hepatitis B elimination in Bangladesh, it is recommended to improve the scope of the national immunisation programme to ensure the vaccination of people at all levels.

## Data Availability

The authors confirm that the datasets used for this study are available on request from the corresponding author.

## Supplementary material

For supplementary material accompanying this paper visit http://doi.org/10.1017/S0950268822000061. click here to view supplementary material
