# Supplementary material for: The prevalence of hepatitis B virus infection in Bangladesh: a systematic review and meta-analysis
Source: Epidemiol Infect. 2022 Feb 14;150:e47. doi: 10.1017/S0950268822000061 (PMC8895722; doi:10.1017/S0950268822000061)
Supplement: Supplementary file 1 [file hygsup.zip › S0950268822000061sup001.docx]

**Supplementary File 2**

**Table S2** Quality assessment of included studies

| **Author, Year of publication** | **Q1** | **Q2** | **Q3** | **Q4** | **Q5** | **Q6** | **Q7** | **Q8** | **Q9** | **Q10** | **Score** |
| --- | --- | --- | --- | --- | --- | --- | --- | --- | --- | --- | --- |
| Laskar MS et al., 1997 | Y | UC | Y | Y | UC | N | N | N | N | Y | 4 |
| Rahman M et al., 1997 | Y | UC | Y | Y | UC | N | N | N | N | Y | 4 |
| Rumi MAK et al., 2000 | Y | N | Y | Y | UC | Y | N | N | N | Y | 5 |
| Gibney L et al., 2001 | Y | N | Y | Y | UC | N | N | N | N | Y | 4 |
| Zaki MH et al., 2003 | Y | UC | Y | Y | UC | Y | N | N | N | Y | 5 |
| Alam MS et al., 2006 | Y | N | Y | Y | UC | Y | N | Y | N | Y | 6 |
| Mahtab M et al., 2008 | Y | UC | Y | Y | UC | Y | N | Y | N | Y | 6 |
| Ashraf H et al., 2010 | Y | UC | Y | Y | UC | N | N | Y | N | Y | 5 |
| Mahtab M et al., 2011 | Y | N | Y | Y | UC | Y | N | Y | N | Y | 6 |
| Rudra S et al., 2011 | Y | UC | Y | Y | UC | Y | N | N | N | Y | 5 |
| Yasmin R et al., 2011 | Y | UC | Y | Y | UC | N | N | N | N | Y | 4 |
| Khan MK et al., 2012 | Y | UC | Y | Y | UC | N | N | N | N | Y | 4 |
| Afroz H et al., 2013 | Y | N | Y | Y | UC | Y | N | N | N | Y | 5 |
| Jobayer M et al., 2016 | Y | N | Y | Y | UC | UC | N | Y | N | Y | 5 |
| Mahtab M et al., 2017 | Y | N | Y | Y | UC | N | N | N | N | Y | 4 |

Y: Yes; N: No; UC: Unclear.
