# Supplementary material for: The prevalence of hepatitis B virus infection in Bangladesh: a systematic review and meta-analysis
Source: Epidemiol Infect. 2022 Feb 14;150:e47. doi: 10.1017/S0950268822000061 (PMC8895722; doi:10.1017/S0950268822000061)
Supplement: Supplementary file 1 [file hygsup.zip › S0950268822000061sup003.docx]

**Supplementary File 1**

**Table S1** Cross-Sectional/Prevalence Study Quality Assessment Forms (AHRQ)

| **Item** | **Yes** | **No** | **Unclear** |
| --- | --- | --- | --- |
| **Q1** Define the source of information (survey, record review) |  |  |  |
| **Q2** List inclusion and exclusion criteria for exposed and unexposed subjects (cases and controls) or refer to previous publications |  |  |  |
| **Q3** Indicate time period used for identifying patients |  |  |  |
| **Q4** Indicate whether or not subjects were consecutive if not population-based |  |  |  |
| **Q5** Indicate if evaluators of subjective components of study were masked to other aspects of the status of the participants |  |  |  |
| **Q6** Describe any assessments undertaken for quality assurance purposes (e.g., test/retest of primary outcome measurements) |  |  |  |
| **Q7** Explain any patient exclusions from analysis |  |  |  |
| **Q8** Describe how confounding was assessed and/or controlled. |  |  |  |
| **Q9** If applicable, explain how missing data were handled in the analysis |  |  |  |
| **Q10** Summarize patient response rates and completeness of data collection |  |  |  |
